# Plastics: Environmental and Biotechnological Perspectives on Microbial Degradation

**DOI:** 10.1128/AEM.01095-19

**Published:** 2019-09-17

**Authors:** Dominik Danso, Jennifer Chow, Wolfgang R. Streit

**Affiliations:** a Department of Microbiology and Biotechnology, University of Hamburg, Hamburg, Germany; University of Bayreuth

**Keywords:** PET, cutinase, microbial plastic degradation, polyamides, polyethylene, polyethylene terephthalate, polypropylene, polystyrene, polyurethane, polyvinylchloride

## Abstract

Plastics are widely used in the global economy, and each year, at least 350 to 400 million tons are being produced. Due to poor recycling and low circular use, millions of tons accumulate annually in terrestrial or marine environments. Today it has become clear that plastic causes adverse effects in all ecosystems and that microplastics are of particular concern to our health. Therefore, recent microbial research has addressed the question of if and to what extent microorganisms can degrade plastics in the environment. This review summarizes current knowledge on microbial plastic degradation. Enzymes available act mainly on the high-molecular-weight polymers of polyethylene terephthalate (PET) and ester-based polyurethane (PUR). Unfortunately, the best PUR- and PET-active enzymes and microorganisms known still have moderate turnover rates. While many reports describing microbial communities degrading chemical additives have been published, no enzymes acting on the high-molecular-weight polymers polystyrene, polyamide, polyvinylchloride, polypropylene, ether-based polyurethane, and polyethylene are known. Together, these polymers comprise more than 80% of annual plastic production. Thus, further research is needed to significantly increase the diversity of enzymes and microorganisms acting on these polymers. This can be achieved by tapping into the global metagenomes of noncultivated microorganisms and dark matter proteins. Only then can novel biocatalysts and organisms be delivered that allow rapid degradation, recycling, or value-added use of the vast majority of most human-made polymers.

## Abstract (precis)

Plastics are widely used in the global economy, and each year, at least 350 to 400 million tons are being produced. Due to poor recycling and low circular use, millions of tons accumulate annually in terrestrial or marine environments. Today it has become clear that plastic causes adverse effects in all ecosystems and that microplastics are of particular concern to our health.

## INTRODUCTION

**FIG 1:**
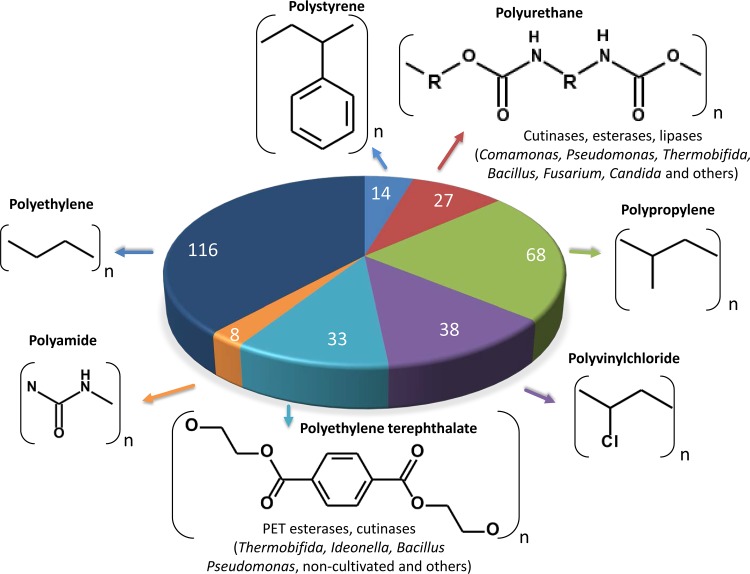
Main synthetic polymers globally produced in 2016. Numbers in the chart indicate the global annual production (millions of tons) of the specified synthetic polymer. Global annual plastic production was extracted from references 1–4, and https://www.plasticsinsight.com/global-pet-resin-production-capacity, https://www.plasticsinsight.com/resin-intelligence/resin-prices/polyamide/, and https://www.plasticsinsight.com/world-plastics-production/. Monomers are depicted above the chart. Indicated are the names of bacterial genera producing verified enzymes with available protein sequences that are known to be involved in the breakdown of the high-molecular-weight polymers (not the additives, plasticizers, etc.). For detailed references on the individual enzymes, refer to the main text. For PA, PE, PS, PVC, and PP, no defined enzymes that act on the polymer have been identified at the level of amino acid or DNA sequences. For enzymes acting on dimers or oligomers and feeding them into the different metabolic pathways, see the main text. For additional structural information on the polymers we refer to ChEBI (https://www.ebi.ac.uk/chebi/init.do).

Altogether, synthetic polymers are produced worldwide at a scale of at least 350 to 400 million metric tons annually (1, 2; see also https://www.plasticsinsight.com/global-pet-resin-production-capacity, https://www.plasticsinsight.com/resin-intelligence/resin-prices/polyamide/, and https://www.plasticsinsight.com/world-plastics-production/). The main polymers that are produced and of importance to our economy are polyurethane (PUR), polyethylene (PE), polyamide (PA), polyethylene terephthalate (PET), polystyrene (PS), polyvinylchloride (PVC), and polypropylene (PP) (Fig. 1). With an increasing production and use of plastics, it is estimated that 5 to 13 million metric tons of plastic enter the ocean every year, with negative consequences for various ecosystems and for the health of humans and animals (1–3). Regarding only the Great Pacific Garbage Patch, more than 1.8 trillion pieces of plastic with an estimated weight of 80,000 tons have so far accumulated, with no end in sight (4–7). While a few reviews have recently been published focusing on the degradation of single types of plastic, only a few articles have addressed plastic degradation on a more global scale, addressing the degradation of several synthetic polymers (8). Therefore, the two main questions addressed in this review are as follows. (i) Which enzymes and microorganisms are currently known to be involved in high-molecular-weight polymer plastic degradation? (ii) What are the future challenges and technologies for identifying better enzymes acting on a highly diverse range of synthetic polymers?

Intriguingly, the currently best-known route of plastic destruction involves exposure to UV light together with mechanical disruption caused by waves and winds or grinding on marine rocks and sediments, which eventually breaks larger plastics into smaller pieces of micro- and nanoplastics (MP, with sizes of <5 mm, and NP, with sizes of <0.1 μm). So-called “weathering” and “photodegradation” are currently considered the main forces for initial depletion of plastics, and they mainly result in a modification of the chemical, physical, and mechanical properties of the plastics (9, 10). The resulting particles have a much larger surface area, which makes them amenable to further degradation (11). Notably, MPs and NPs are a concern to our health, as it is expected that they enter the food chain and end up in our intestines (12, 13). The fate of MPs or NPs in human or animal intestines has yet to be determined.

Therefore, removal of plastics from the environment using microbial enzymes has been a focus of recent research. The main challenge is that marine and terrestrial displaced plastics are highly stable and durable. Plastics have mainly been introduced since the 1960s and, given the relatively few decades since these human-made polymers became available, nature has only had a very short time to evolve highly active enzymes. Besides, many different types of plastics accumulate in the environment, and many of the frequently used plastics are mixtures containing additional solubilizers and other chemical agents to alter the mechanical and physical properties. These compounds are further targets for microbial biodegradation but may also interfere with degradative enzyme activities. It is assumed that the larger polymers are initially degraded by secreted exoenzymes into smaller subunits (multimers, dimers) that can be incorporated into the microbial cells. Once in the cells, either the oligomers or the degradation products of these are funneled through the classical degradation pathways to yield energy and/or serve as building blocks for catabolism or metabolism.

**FIG 2:**
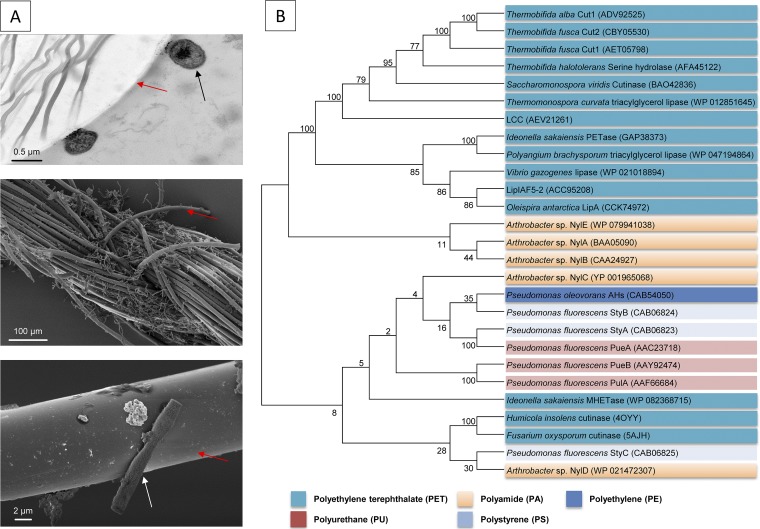
(A) Electron microscopic images of Comamonas sp. strain DDHH 01 attached and hydrolyzing PET fibers. *Comamonas* sp. DDHH 01 was isolated from a sewage enrichment culture. Red arrows indicate PET fibers. Black and white arrows indicate bacterial cells. (Top) Transmission electron microscopy image of a PET fiber with attached *Comamonas* sp. cells. (Middle) Scanning electron microscope image of PET yarn with microcolonies. (Bottom) Closeup of a single cell on the surface of a single PET fiber. (B) Topology of a neighbor-joining tree containing representative sequences of most of the currently known synthetic polymer- or oligomer/monomer-degrading enzymes. The tree is based on amino acid sequence homologies. Overall, 27 known functional and verified enzymes were included in this alignment. This represents the majority of the currently known and biochemically characterized enzymes. PET hydrolases represent the largest fraction of known and studied enzymes. The alignment was calculated using T-Coffee in accurate mode (124). The tree was calculated with Molecular Evolutionary Genetics Analysis version 6 (MEGA6) (125) and is not rooted. A similarity and identity matrix for all included sequences, together with their accession numbers, is provided in the supplemental material (Table S1).

Within this framework, this review summarizes the main findings on microbial degradation of the polymers listed above. The chemical structures and some properties of these polymers are described in each of the following subsections. For a first overview on enzymes and microbes acting on the different plastics, see Fig. 1 and 2.

In general, it is believed that the microbial degradation of human-made polymers is a very slow process. This high resistance mainly stems from the high molecular weight of the fiber, the strong C-C bonds, and the extremely hydrophobic surface, which is very difficult to attack by enzymes. Notably, polymers are high-molecular-weight molecules, and they have amorphous and crystalline forms, which have different levels of degradability.

## POLYMERS AND MICROBIAL DEGRADATION

### Polyethylene terephthalate.

Polyethylene terephthalate (PET) is mainly used for production of PET bottles, PET foil, and fibers in the textile industry. PET is a polar, linear polymer of repeating units of the aromatic terephthalic acid and ethylene glycol. The PET monomer is designated bis(2-hydroxyethyl) terephthalate (BHET) (14). PET is a thermoplast and partly crystalline. The annual production of PET exceeded 30 million tons in 2017 (https://www.plasticsinsight.com/global-pet-resin-production-capacity).

Currently, only a few bacteria and fungi have been described for the partial degradation of PET to oligomers or monomers (8). All known PET hydrolases have relatively low turnover rates. Intriguingly, the trait for PET degradation appears to be limited to a few bacterial phyla, and most bacterial isolates with the potential for PET degradation are members of the Gram-positive phylum Actinobacteria (15). The best characterized examples originate from the genera Thermobifida and Thermomonospora (16–23). The enzymes involved in the degradation (e.g., PET hydrolase and tannase, MHETase) are typical serine hydrolases, e.g., cutinases (EC 3.1.1.74), lipases (EC 3.1.1.3), and carboxylesterases (EC 3.1.1.1). These enzymes possess a typical α/β-hydrolase fold, and the catalytic triad is composed of a serine, a histidine, and an aspartate residue (18, 24). They can also contain several disulfide bonds caused by cysteine residues, which promote thermal stability and specific binding to PET, as shown by the example of PETase from Ideonella sakaiensis 201-F6 (25).

Also, for the bacterium I. sakaiensis, usage of PET as a major energy and carbon source has been described (25). In addition to the PET hydrolase, the *I. sakaiensis* genome codes for a second enzyme that appears to be unique so far and which shares high similarity to the group of tannases, capable of degrading mono(2-hydroxyethyl) terephthalic acid. PET hydrolase as a secreted enzyme produces the intermediate mono(2-hydroxyethyl) terephthalic acid (MHET). MHET is internalized by the cell and hydrolyzed by MHETase. The resulting monomers are then used for bacterial metabolism. *I. sakaiensis* is affiliated with the phylum Betaproteobacteria and belongs to the order Burkholderiales.

The *I. sakaiensis* PETase three-dimensional (3D) structure was elucidated recently (26). The overall structure most resembles the structures of cutinases. Austin et al. showed that a double mutation (S238F/W159H), which narrows the active site of the enzyme and makes the protein even more like a cutinase resembling the enzyme from Thermobifida fusca, leads to an improved variant. The majority of the functionally verified PET hydrolases contain a C-terminal disulfide bond, promoting thermal and also kinetic stability (27–29). The only exception from this so far is a *para*-nitrobenzylesterase from Bacillus subtilis (30). An additional disulfide bond can be found in *I. sakaiensis* PETase, as well as in structural models of the functionally tested PET hydrolases described by Danso et al. (31). The structural data indicate that PETases bind the polymer with the hydrophobic surface and the substrate-binding cleft. In total, 4 MHET moieties are bound to the protein (one to subsite I and three to subsite II), whereby the ester bond to be cleaved is located between both subsites next to the catalytic serine. The MHETase from *I. sakaiensis* that further hydrolyzes MHET to ethylene glycol and terephthalic acid has been recently crystallized ligand free (2.05 Å) and with a nonhydrolyzable MHET analogue bound (2.1 Å). The enzyme possesses a lid domain that almost exclusively confers substrate specificity and activity toward MHET, with a *k*_cat_ of 11.1 ± 1.4 s^−1^ (32).

While the *I. sakaiensis* enzymes are the best-studied models, other enzymes and organisms have been identified as potent PET degraders. Currently, four enzymes from *Thermobifida* species, one from Saccharomonospora, and one from the phylum Thermomonospora are known to act on PET. These actinobacterial enzymes are often Ca^2+^-dependent, especially in terms of their thermal stability (33), and they are partially inhibited by their released hydrolysis products MHET and BHET (33). Therefore, efforts have been made to overcome this limitation; one approach lies in the combination of polyester hydrolases with other enzymes to improve substrate binding and catalytic properties (26, 34, 35).

Besides the actinobacterial PET hydrolases, fungal cutinases showed activity on PET substrates as well. The most prominent examples are cutinases of the phyla Fusarium and Humicola. The latter was also used together with the lipase CalB from Candida antarctica in order to circumvent the previously mentioned product inhibition by BHET and MHET (34). While CalB completely converted to terephthalic acid, the *Humicola*-derived enzyme was limited in the last reaction step and accumulated the intermediate MHET.

Complementary to the above outlined activity-based approaches, a hidden Markov model (HMM) motif-based large-scale global search of existing genome and metagenome databases has been developed for the presence of potential PET hydrolases (31). Using this approach, >800 potential PET hydrolases were identified in bacterial and archaeal genomes and metagenomes, and several enzymes were functionally verified (e.g., PET2, PET4, PET6, and PET12). These findings imply that PET hydrolase-encoding genes are globally distributed in marine and terrestrial metagenomes (31).

Using an *in silico* genome mining approach, a cutinase from Pseudomonas pseudoalcaligenes (PpCutA) and a putative lipase from Pseudomonas pelagia (PpelaLip) were identified as potential enzymes acting on polyesters in general. Further experimental work using recombinant enzymes of PpCutA and PpelaLip verified the hydrolytic activities of both enzymes on different types of polyesters, including the hydrolysis of polyoxyethylene terephthalate (36). In their study, the authors used structurally different ionic phthalic acid-based polyesters with an average molecular weight ranging from 1,770 to 10,000 g/mol and semicrystalline polyesters with crystallinity below 1% to test and verify the microbial degradation. Notably, the identified organism belongs to a biotechnologically important novel species within the genus Pseudomonas, which was designated Pseudomonas pertucinogena (37).

In addition to the metagenome-derived PET esterases described above, colleagues recently reported on the functional screening of metagenomes and the characterization of selected enzymes. Among those were the metagenome-derived esterases MGS0156 and GEN0105, which hydrolyzed polylactic acid (PLA) and polycaprolactone, as well as bis(benzoyloxyethyl)-terephthalate. For MGS0156, 3D structural data at 1.95 Å indicate a modified α/β-hydrolase fold with a lid domain and a highly hydrophobic active site (38). The closest homologue to MGS0156 is an enzyme from Desulfovibrio fructosivorans with 70% sequence similarity.

In summary, PETases represent the best-explored and -studied class of enzymes with respect to the hydrolysis of synthetic polymers.

### Polyurethanes.

Polyurethanes (PUR) can be synthesized by using different polyether or polyester polyols. PUR is a polymer of organic units connected by carbamate. The additional incorporation of aromatic ring structures has further impact on the physical and chemical properties of the polymer. PUR is a widely used synthetic polymer for the production of foams, insulation materials, textile coatings, and paint to prevent corrosion (39). With over 27 tons produced annually (2), it ranks fifth among the most often produced synthetic polymers.

To date, only bioactivities that act on the ester-based PUR have been reported (40, 41). Biodegradation was achieved by either bacteria or fungi. With respect to bacteria capable of degrading PUR, Gram-negative Betaproteobacteria from the genus *Pseudomonas* have been most frequently linked with PUR activities. One of the first enzymes identified to act on PUR was the PueB lipase from Pseudomonas chlororaphis (42, 43). This organism codes for at least one additional enzyme active on PUR, which was designated PueA (44). Both enzymes are lipases; PUR is degraded by the secreted hydrolases, and the degradation is tightly regulated. Their respective genes are part of a larger gene cluster encompassing seven open reading frames (ORFs) (45). Pseudomonas protegens strain Pf-5 uses a similar mechanism to degrade dispersions of the polyester PUR. In this strain, however, it was shown that PUR degradation is tightly regulated by mechanisms of carbon catabolite control and that both lipase genes, *pueE* and *pueB*, appear to be essential for growth on PUR dispersions (46). In a similar manner, Pseudomonas putida was reported to degrade PUR at relatively high rates (47). The bacterium needed 4 days to grow and consume the added colloidal PUR. Yet another example comes from Comamonas acidovorans TB-35. This strain produces a PUR-active enzyme that is an esterase and which was designated PudA (48, 49). PudA shows a hydrophobic PUR-surface-binding domain and a distinct catalytic domain, and its surface-binding domain is considered to be essential for PUR degradation. PudA acts as a 62-kDa monomer, and it releases diethylene glycol and adipic acid at an optimum temperature of 45°C and an optimum pH of 6.5.

Within this context, it is perhaps notable that often enzyme activities that are reported are based on clearing zones in agar plates. However, these assays are not fully reliable. For instance, different enzymes from *Pseudomonas* spp. and *Bacillus* spp. showed significant esterase activities and partially or even completely cleared plates containing colloidal PUR. However, only the *Pseudomonas* sp. lipase significantly degraded the added PUR based on nuclear magnetic resonance (NMR) and infrared (IR) data (50). Furthermore, there is strong evidence that some B. subtilis and Alicycliphilus sp. isolates are able to degrade PUR (51–53).

In a recent publication, Schmidt and colleagues reported on microbial degradation of PUR (i.e., Impranil DLN). The authors of this study employed the known polyester hydrolases LC-cutinase, TfCut2, Tcur1278, and Tcur0390 in their assays and observed significant weight loss of the tested foils when incubated for extended time periods (200 h) at a temperature of 70°C (54). The observation that cutinases, otherwise known to degrade polyethylene terephthalate, also act on PUR could be attributed to the promiscuous nature of the *Thermobifida*-derived cutinases. Recent research on promiscuity of enzymes implies that lipolytic enzymes such as cutinases are very often highly promiscuous and can convert up to 78 different substrates (55).

While the list of PUR-active bacteria is steadily increasing, a larger number of fungi have also been reported to degrade polyurethane (41). Notably, the authors of that study identified a 21-kDa metallo-hydrolase from Pestalotiopsis microspora as a responsible enzyme in PUR degradation.

Additional studies identified Fusarium solani, Candida ethanolica (56), and Candida rugosa (57) as PUR degraders. While for C. rugosa, a lipase has been identified as the key enzyme involved in PUR metabolism, no enzymes were yet identified for *C. ethanolica* and *F. solani*. Other fungi reported belong to the Cladosporium cladosporioides complex, including the species Cladosporium pseudocladosporioides, Cladosporium tenuissimum, Cladosporium asperulatum, and Cladosporium montecillanum, and three others were identified as Aspergillus fumigatus, Penicillium chrysogenum (58), and Aspergillus flavus (59). In the case of A. flavus, it is assumed that secreted esterases are responsible for the degradation. However, no defined enzyme has yet been linked to the observed activities. In a similar study, it was recently reported that Aspergillus tubingensis colonizes PUR and acts on the surface of films made of PUR. However, no enzyme was linked with the PUR activities (60).

It is noteworthy that the above-mentioned PUR-active enzymes and organisms were all acting on ester-linked PUR. However, to the best of our knowledge, no enzymes have yet been described acting on polyurethane ethers.

### Polyethylene.

Polyethylene (PE) consists of long-chain polymers of ethylene, and it is produced as either high-density (HD-PE) or low-density (LD-PE) polyethylene. PE is chemically synthesized by polymerization of ethane and is highly variable, since side chains can be obtained depending on the manufacturing process. Such modifications mainly have influence on crystallinity and molecular weight. The polymer is most frequently used in the packaging industry as one of the main packaging materials, and more than 100 million tons of PE are produced globally per year (2, 61) (Fig. 2).

Possible PE degradation has been affiliated with a surprisingly large number of bacterial genera. Among those were Gram-negative species affiliated with the genera *Pseudomonas*, Ralstonia, and Stenotrophomonas but also many Gram-positive taxa (e.g., Rhodococcus, Staphylococcus, Streptomyces, Bacillus, and others) (see references in Sen and Raut [62] and Restrepo-Florez et al. [63]). In addition, fungal genera affiliated with assumed PE degradation were reported; these included Aspergillus, Cladosporium, Penicillium, and others (see references in references 62, 63, and 64–69). In addition, a few studies linked the PE-degrading microbes with the complex gut microbiomes of invertebrates (70, 71).

It is notable that in almost all the above-mentioned studies on PE-degrading microorganisms, the authors reported on degradation of the polymers using commercial polymers that possibly contained chemical additives, and degradation was determined by measuring weight loss and by Fourier transform infrared spectroscopy (FTIR). Since weight loss and surface structure changes are most likely attributed to the degradation of chemical additives, which often make up a significant fraction of the polymer, the results in these studies need to be verified using more advanced technologies. None of these studies reveled biochemical mechanisms and enzymes involved in PE breakdown. Within this framework, a more recent publication identified a *Penicillium*-derived laccase as potentially involved in PE breakdown (72). Unfortunately, no detailed biochemical characterization was performed, and no sequence of the protein or the corresponding gene was deposited.

### Polyamide.

Polyamide (PA) is a polymer of repeating units of aliphatic, semiaromatic, or aromatic molecules linked via amide bonds. Since the monomers for making this polymer can be very versatile, there are many different types of synthetic polyamides, with the most popular being nylon and Kevlar. Synthetic polyamides are mainly used in textiles, automotive applications, carpets, and sportswear (73).

Remarkably, proteins as well as natural silk are polyamides *per se*. Based on this, it should be expected that nature has evolved enzymes that act on these nonnative polymers. However, to date, there is no microorganism known that is able to fully degrade the intact high-molecular-weight polymer. In contrast, several studies are available on bacteria acting on either linear or cyclic nylon oligomers with rather short chain lengths. In one of the first studies, different bacteria were described to grow on various oligomers derived from nylon production (74). In wastewater of nylon factories, 8-caprolactam, 6-aminohexanoic acid, 6-aminohexanoic acid cyclic dimer, and 6-aminohexanoic acid oligomers accumulate. These compounds can serve as the carbon and nitrogen source for specially adapted bacteria. One of the first bacteria described growing on these mixtures of oligomers was Flavobacterium sp. strain KI72, which was later renamed Achromobacter guttatus KI72 and then recently named Arthrobacter sp. strain KI72 (74, 75). Nylon oligomer-degrading *Arthrobacter* isolates code in their genomes for different hydrolases and several aminotransferases involved in the initial degradation of the oligomers and the subsequent metabolism. In the case of strain KI72, the respective genes are located on an accessory plasmid, pOAD2 (76–78).

Three main enzymes are essential for the initial hydrolysis of cyclic and linear 6-aminohexanoate oligomers. The first one is a cyclic-dimer hydrolase (NylA), the second a dimer hydrolase (NylB), and the third an endo-type oligomer hydrolase (NylC). NylC is a typical esterase, but its 3D structure also reveals motifs with β-lactamase folds (79–87). Once the oligomers are hydrolyzed, the monomers are metabolized by different aminotransferases. The draft genome of *Arthrobacter* sp. KI72 carries, among others, two genes, designated *nylD1* and *nylE1*, that are responsible for the secondary 6-aminohexanoate metabolism. The 6-aminohexanoate aminotransferase (NylD1) catalyzes the reaction of 6-aminohexanoate to adipate semialdehyde. It uses α-ketoglutarate, pyruvate, and glyoxylate as amino acceptors and generates glutamate, alanine, and glycine, respectively. The reaction relies on pyridoxal phosphate as a cofactor. The second enzyme, the adipate semialdehyde dehydrogenase (NylE1), catalyzes the reaction, leading from adipate semialdehyde to adipate. This enzyme requires NADP^+^ as a cofactor and is an oxidoreductase (88, 89).

More recently, diverse marine bacteria were reported to act on nylon. The authors of this study reported a significant weight loss over a time period of 3 months. In their study, Bacillus cereus, Bacillus sphaericus, Vibrio furnissii, and Brevundimonas vesicularis were identified as potential nylon degraders (90). The genes and enzymes associated with the nylon degradation, however, remain to be identified, and the possibility cannot be excluded that the weight loss observed was primarily linked to the degradation of chemical additives, as outlined above.

Rather than using the synthetic polymer, Oppermann and colleagues reported on 12 bacterial species capable of degrading the natural polymer poly-γ-glutamic acid. The high-molecular-weight polymer is synthesized by many Gram-positive bacteria as a major component of capsules and slime. In contrast to the synthetic polymer, however, it is a water-soluble molecule and is thus more easily accessible to microbial degradation (91).

The only enzyme that has so far been reported to act on high-molecular-weight nylon fibers was classified as a manganese-dependent peroxidase and originated from a white rot fungus. The activity of the native and purified enzyme, however, differed from that of lignolytic enzymes. Nylon-degrading activity was quantified by measuring the structural disintegration of nylon-66 membranes. The enzyme had a molecular weight of 43 kDa and was dependent on the presence of lactate and other alpha-hydroxy acids. Unfortunately, no gene or protein sequence was determined (92).

While the first reports were published in 1965 stating that, among others, Pseudomonas aeruginosa is able to convert oligomeric nylon, further studies have confirmed that P. aeruginosa and evolved strain PAO1 are able to efficiently degrade 6-aminohexanoate linear dimers (74, 93). The main enzymatic activities were assigned to a 6-aminohexanoate cyclic-dimer hydrolase and a 6-aminohexanoate dimer hydrolase. Other *Pseudomonas* species have, however, also been reported to utilize 6-aminohexanoate-dimers as a sole carbon and nitrogen source (94).

### Polystyrene.

Polystyrene (PS) [poly(1-phenylethene)] polymer consists of styrene monomers. PS is a widely used synthetic polymer for packaging industries but many daily use articles (CD cases, plastic cutlery, petri dishes, etc.) are also produced from this polymer (95). In 2016, about 14 million tons were produced (https://www.plasticsinsight.com/global-pet-resin-production-capacity).

Unfortunately, there is no enzyme known today that can degrade the high-molecular-weight polymer. However, a first report was published recently by Krueger and colleagues on the identification of brown rot fungi able to attack polystyrol by employing hydroquinone-driven Fenton reactions. In this preliminary study, Gloeophyllum striatum DSM 9592 and Gloeophyllum trabeum DSM 1398 caused substantial depolymerization after 20 days of incubation. The most active Gloeophyllum strains caused almost 50% reductions in molecular weight (96). In an earlier study, the white rot fungi Pleurotus ostreatus, Phanerochaete chrysosporium, and Trametes versicolor and the brown rot fungus Gloeophyllum trabeum were affiliated with the depolymerization of polystyrene when coincubation together with lignin was performed (97). While these are first and promising reports on the degradation of the high-molecular-weight polymer, the enzymes involved in the depolymerizing reaction remain to be elucidated. As already outlined above, weight loss may have been caused by the degradation of chemical additives.

Similarly, several bacteria have been reported to form either alone or as members of consortium biofilms on polystyrene films and particles, thereby degrading the polymer. In these studies, mainly weight loss has been assayed. Unfortunately, in none of these studies were enzymes linked to the assumed depolymerization (98, 99).

While not a single bacterium is known to degrade the polymer, a larger number of bacterial genera that are capable of metabolizing the monomer styrene as a sole source of carbon are known. The biochemistry of styrene metabolism is well understood, and for more detailed reviews, see references 98 and 100–103 and references therein. Styrene degradation in bacteria is well studied in *Pseudomonas*, Xanthobacter, Rhodococcus, Corynebacterium, and others. It appears to be a widespread metabolism. Under aerobic conditions, styrene is oxidized by two different pathways, namely, (i) attacking the vinyl side chain and (ii) a rather unspecific aromatic ring, thereby forming primarily the intermediates 3-vinylcatechol, phenylacetic acid, and 2-phenylethanol. These intermediates are channeled into the Krebs cycle after ring cleavage. The degradation of the vinyl side chain involves the action of three key enzymes, a styrene monooxygenase, a styrene oxide isomerase, and a phenylacetaldehyde dehydrogenase (104). The styrene monooxygenase attacks the vinyl side chain to release epoxystyrene, which is then subjected to isomerization to form phenylacetaldehyde. The latter is oxidized to phenylacetic acid though the involvement of a dehydrogenase. In P. putida, the phenylacetic acid is activated to phenylacetyl-coenzyme A (CoA) and then subjected to β-oxidation to yield acetyl-CoA, which is directly fed into the Krebs cycle. The respective genes for side-chain oxygenation are frequently located in a single conserved gene cluster, often designated *styABC(D)* (105). Thereby, the *styA* and *styB* genes code for the styrene monooxygenase complex. The styrene monooxygenase is a two-component flavoprotein that catalyzes the NADH- and FAD-dependent epoxidation of styrene to styrene oxide. StyA is the actual monooxygenase, and StyB functions as flavin adenine dinucleotide (FAD) reductase, which transfers the electrons from NADH to FAD^+^ to supply StyA with the required electrons (106). The *styC* gene codes for the styrene isomerase (107), and *styD* is a phenylacetaldehyde dehydrogenase gene (108). The expression of the conserved cluster is regulated through either a two-component regulatory system or LysR-type regulators (109–111).

The direct ring cleavage of styrene is initiated by a dihydroxylation of the aromatic ring. This reaction is catalyzed by a 2,3-dioxygenase and followed by a 2,3-dihydrodiol dehydrogenase. The two key products that are formed are styrene *cis*-glycol and 3-vinylcatechol. The latter can then be degraded by subsequent meta- or orthocleavage to form acrylic acid, acetaldehyde, and pyruvate. The pathway is rather unspecific for the general degradation of various aromatic compounds, such as phenol or toluene (100–102).

The produced phenylacetaldehydes are of interest to different industries, as they can be considered building blocks for the production of different fine chemicals or pharmaceutical compounds. They can serve as the starting material to synthesize fragrances, flavors, pharmaceuticals, insecticides, fungicides, or herbicides (112). Recent studies have also shown that Pseudomonas putida, Rhodococcus zopfii, and other Gram-negative species can convert polystyrene (i.e., styrene oil) into the biodegradable polymer polyhydroxyalkanoate or other valuable compounds. The approach involves as a first step the pyrolysis of polystyrene to styrene oil. The styrene oil is then converted in a second step to polyhydroxyalkanoate or other compounds. While the overall concept of this two-step process is intriguing, it may not be feasible on a large scale, as the pyrolysis is a process that runs at 520°C and this is energetically very demanding (113–115).

### Polyvinylchloride and polypropylene.

Polyvinylchloride (PVC) and polypropylene (PP) are both important polymers produced at higher levels than the above-named polymers. PVC is the third most frequently produced polymer, and only PE and PP are produced at higher levels. PVC is composed of repeating chloroethyl units and PP of repeating units of propane-1,2-diyl units (116, 117). In sharp contrast to their huge global production rate, hardly any reliable information is available on microbial degradation of both of these important polymers. Only a very few reports that describe the degradation of the polymers based on weight loss and using mixed species microbial communities have been published (118, 119). However, it is likely that these reports were in part misled by the degradation of the chemical additives rather than the polymer. Consequently, no defined enzymes or pathways that are responsible for the degradation of either of these two high-molecular-weight polymers are known.

## MICROBIOMES OF INVERTEBRATES AS POSSIBLE SOURCES OF PLASTIC-DEGRADING BACTERIA

Recently, it was reported that invertebrates can degrade different plastics (70, 71, 120–123). While these studies demonstrated that the insects perform a mechanical grinding and shredding of the plastics, it has been critically discussed if, and to which extent, the microbiomes associated with the different insects are capable of truly degrading the synthetic polymers. In one of those studies, Yang and colleagues provided convincing evidence that Tenebrio molitor L. (mealworms) digested Styrofoam. The larvae lived over a month when fed on the Styrofoam. Within a 16-day period, nearly 50% of the ingested Styrofoam carbon was converted into CO_2_, and the residual Styrofoam was found in the feces. Labeling studies using α-^13^C- or β-^13^C-labeled polystyrol implied that the carbon compound was preferentially used to build lipids (71). One of the earliest reports on insects digesting plastics came from caterpillars. In 2017, a Spanish team reported on the fast biodegradation of PE by larvae of the wax moth (Galleria mellonella). The authors of this study presented evidence that larvae of the wax moth produced holes in PE films with considerable speed (120). The findings of this study were critically discussed later on, as the occurrence of ethylene glycol as well as the correct usage of the FTIR method could not be immediately verified (121). Further work by a Chinese and United States-based research team identified *Bacillus* sp. strain YP1 as the polyethylene-degrading bacterium responsible for PE degradation in Indian mealworms (70, 122). A related study from the same group identified bacteria affiliated with the genera *Citrobacter* and Kosakonia as main degraders for PE and PS in the guts of *Tenebrio molitor* (123).

Thus, grinding of larger plastic pieces into smaller parts might offer a solution in that it increases the surface area and thereby allows microorganisms to better attach to the surfaces.

## FUTURE CHALLENGES IN MICROBIAL PLASTIC DEGRADATION RESEARCH

The diversity of known enzymes and microbes acting on synthetic polymers is still rather limited. Therefore, future work has to address the identification of organisms acting on the most dominant polymers. The main bottleneck lies in the initial breakdown of high-molecular-weight and highly robust polymers and their crystalline structures. Furthermore, the implementation of enzymes in processes that would allow the degradation of plastic polluting environmental niches is a challenge for future generations of microbiologists. Since current cultivation technologies have not yet resulted in the identification of highly active enzymes for most plastics, the diversity of noncultivated microorganisms (i.e., global metagenomes) and the so-called dark matter proteins offer a promising source for the identification of such biocatalysts. Thus, the further development of smart search algorithms for mining metagenome data sets is certainly a rewarding task. In parallel, the setup of reliable function-based assays for the detection of high-molecular-weight-polymer-active enzymes is important as well.

Since commercially available polymers and films thereof are often used as substrates, they contain additives, plasticizers, and other biodegradable impurities (for example, phthalates), which are much more easily broken down than the actual backbone. This therefore interferes with the results and frequently leads to the identification of false positives. Thus, the overall methodology linked to the analysis of microbial plastic degradation needs to be standardized and optimized.

Similarly, the development of cellulosome-like structures (i.e., “plastosomes”) in microbes to attack intact and crystalline fibers would certainly be a worthwhile project. Along these lines, the simple development of highly active enzymes for textile industries could already significantly reduce annual plastic pollution and would perhaps be one of the more realistic short-term goals.

Furthermore, using synthetic biology to generate microorganisms that would produce high-value compounds from plastic waste is a future challenge and would contribute to an improved circular use of plastics. Monomers and oligomers formed after the degradation could be used to build value-added products or even new (biodegradable) polymers.

Lastly, obtaining plastic-active enzymes and implementing them in the production of true biopolymers is a highly rewarding research task and would significantly reduce our global plastic problem.

## Supplementary Material

Supplemental file 1
